# Rituximab and Intravenous Immunoglobulin (IVIG) for Refractory Eosinophilic Fasciitis: A Case Report

**DOI:** 10.1002/ccr3.71766

**Published:** 2026-01-04

**Authors:** Maryam Sahebari, Motahhareh Karimoddini, Naser Tayyebi Meibodi, Sajad Ataei Azimi, Shadan Tafreshian

**Affiliations:** ^1^ Rheumatic Diseases Research Center, Mashhad University of Medical Sciences Mashhad Iran; ^2^ Internal Medicine Department, Faculty of Medicine, Mashhad University of Medical Sciences Mashhad Iran; ^3^ Department of Pathology Mashhad University of Medical Sciences Mashhad Iran; ^4^ Hematology and Oncology Department, Faculty of Medicine, Mashhad University of Medical Sciences Mashhad Iran

**Keywords:** eosinophilic fasciitis, intravenous immunoglobulin, refractory eosinophilic fasciitis, rituximab

## Abstract

Eosinophilic fasciitis (EF) is a rare condition with an unknown cause. This case study showed that a 40‐year‐old man with EF did not respond to standard treatments but improved after receiving rituximab and intravenous immunoglobulin (IVIG). Further studies are needed to confirm rituximab's effectiveness and long‐term safety for EF.

## 1 Introduction

Eosinophilic fasciitis (EF), also known as Shulman's syndrome, is a rare connective tissue disorder characterized by inflammation and thickening of the fascia. This condition is often associated with peripheral eosinophilia, and its exact cause remains unknown. Symptoms of EF primarily include skin hardening and stiffness, particularly in the extremities [1, 2]. Standard treatment typically consists of high‐dose corticosteroids and immunosuppressive agents, such as methotrexate. However, some patients do not respond effectively to these therapies, which poses a significant challenge for management [3]. Research is ongoing to better understand the role of B cells in the pathogenesis of EF. Rituximab, a monoclonal antibody that targets CD20‐positive B cells, has been used successfully to treat various autoimmune diseases. Its positive outcomes in other conditions suggest that it could be a new treatment option for EF patients who do not respond to standard treatments [4, 5]. In this case report, a patient with EF resistant to corticosteroids and methotrexate demonstrated mild clinical improvement after receiving treatment with rituximab. This suggests that rituximab may be an effective treatment strategy for managing refractory EF.

## 2 Case History and Physical Examinations

### 2.1 History/Examination

A 40‐year‐old professional bodybuilder and fireman with no medical history and no drug and supplement use developed severe myalgia and arthralgia 5 h after long‐distance mountain climbing with a 30‐kg backpack, and 48 h later, night fever and sweating, non‐productive cough and fatigue were added to his symptoms. The patient went to the emergency department and underwent a workup. In the initial examination, the vital signs were stable and the temperature was 37.9°C. Wheezing was heard in lung auscultation. Other examinations were normal.

### 2.2 Investigations, Diagnosis and Treatment

The findings of the laboratory tests are shown in Table 1. Due to hyper eosinophilia (eosinophil count: 3944/mm^3^), spirometry and lung Computed Tomography (CT) were performed, which were normal. Additional laboratory tests were requested, which were all negative (Table 1). Due to hyper eosinophilia, a hematology consultation was requested. Given the platelet count of 650 × 10^3^/μL and suspicion of underlying thrombocythemia, bone marrow aspiration and biopsy were performed, and JAK2, MPL, and CALR genes were evaluated, which were negative. A molecular study was also conducted, and FIP1L1‐PDGFRA fusion was normal. However, weight loss continued, and after 2 months, the patient had lost 11 kg. For this reason, the hematologist decided to prescribe low‐dose corticosteroids as a 2‐week trial, which improved myalgia and B symptoms, as well as inflammatory markers and eosinophil count (1092/mm^3^) but did not affect the severity of weakness. CT scans of the neck, chest, abdomen, and pelvic with and without contrast were performed to check for malignancy, all of which were normal.

**TABLE 1:** The results of laboratory tests.

Parameter	Lab value	Reference range
WBC	11.6 × 10^3^/μL	3.5–11 × 10^3^/μL
PMN	34%	40%–60%
Lymph	21%	20%–40%
Eos	34%	1%–4%
Hb	13 g/dL	11.5–17.1 g/dL
MCV	85 fl	78–100 fl
Plt	650 × 10^3^/μL	120–450 × 10^3^/μL
ESR 1st hr	34 mm/h	< 20 mm/h
CRP	69 mg/L	0–5 mg/L
RF titer (mg/dL)	3 mg/dL	0–14 mg/dL
ANA	0.4 U/mL	< 1 U/mL
FANA	1/40	< 1/80
ANA Profile (IgG)	Negative	—
Anti dsDNA (IgG)	35 IU/mL	< 80 IU/mL
ANCA	8 AU/mL	< 19 AU/mL
IgG	710 mg/dL	600–1500 mg/dL
IgG4	10 mg/dL	2.4–121 mg/dL

Abbreviations: ANA (Antinuclear Antibody), ANCA: antineutrophil cytoplasmic antibodies, anti‐dsDNA: anti‐double‐stranded DNA, CRP: C‐reactive protein, EOS: eosinophils, ESR: Erythrocyte sedimentation rate, FANA (fluorescent antinuclear antibody), Hb: hemoglobin, IgG: Immunoglobulin G, IgG4: Immunoglobulin G4‐related disease, Lymph: lymphocytes, MCV: mean corpuscular volume, Plt: platelet, PMN: Polymorphonuclear leukocytes, RF: Rheumatoid factor, WBC: white blood cells.

**FIGURE 1:**
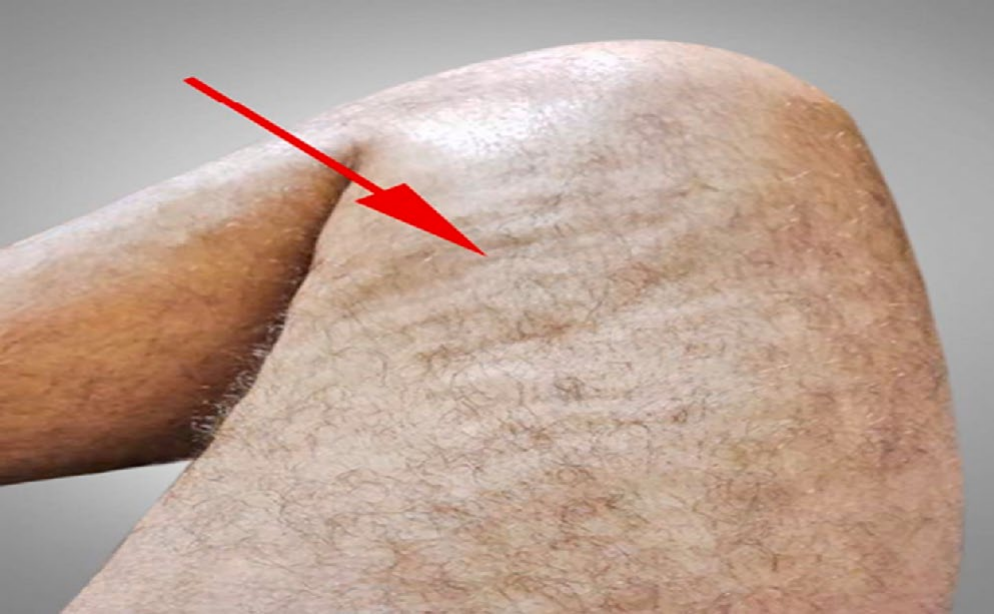
Peau d'orange appearance.

**FIGURE 2:**
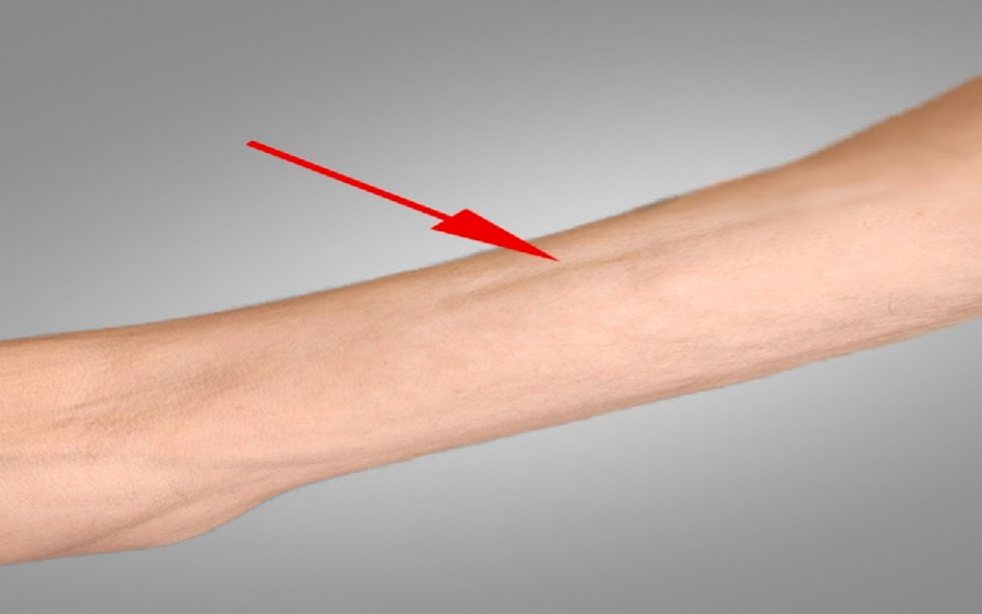
Groove sign.

**FIGURE 3:**
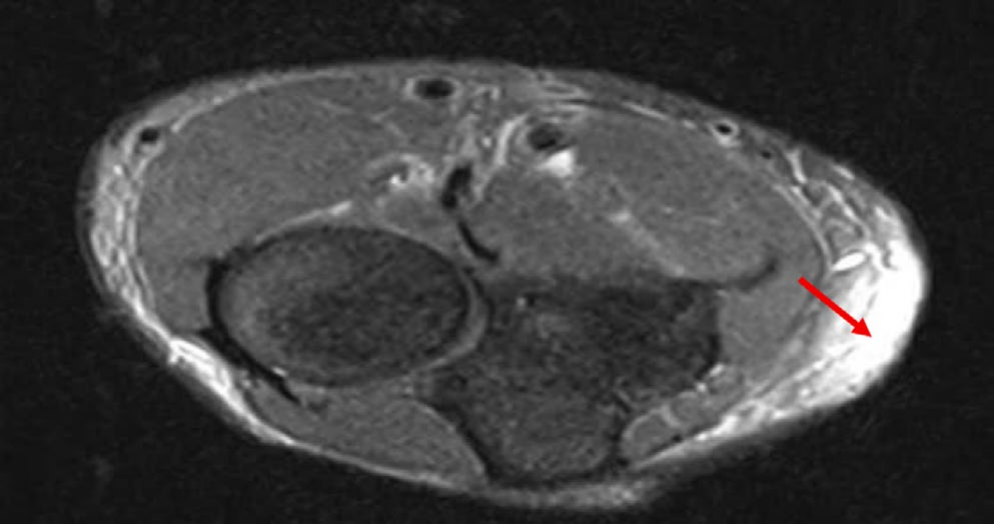
Elbow Magnetic Resonance Imaging (MRI). Edema in the subcutaneous tissue, especially on the medial side of the elbow, extending to the proximal forearm.

**FIGURE 4:**
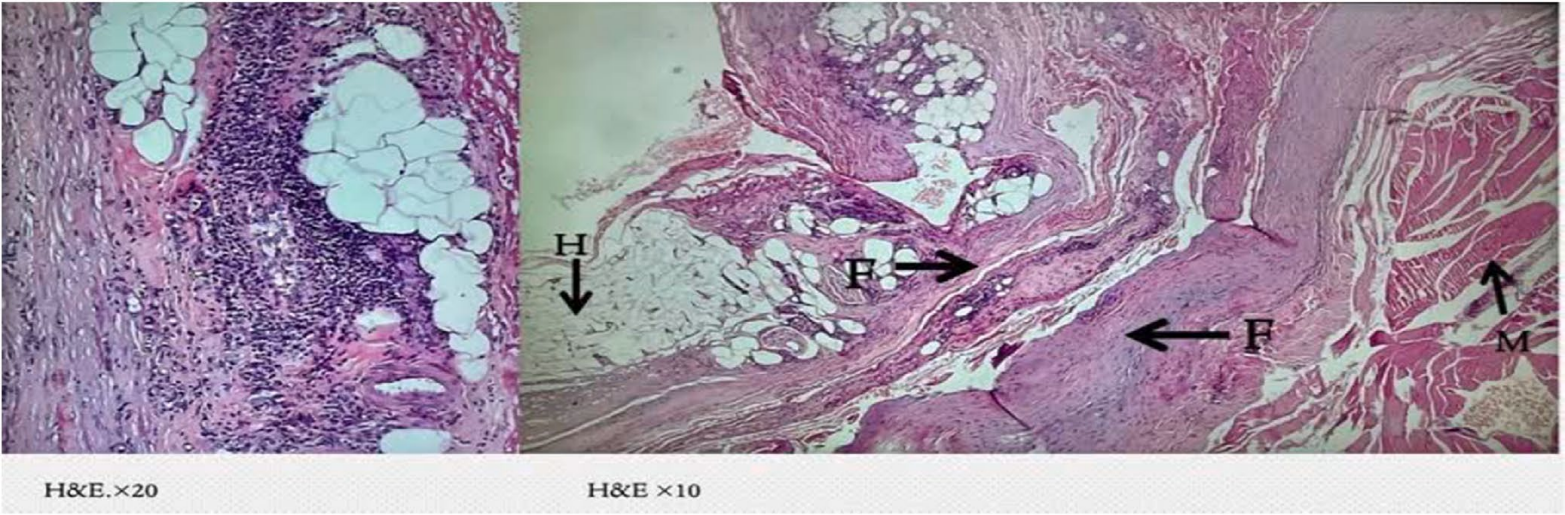
Right arm and forearm skin with muscle and fascia biopsy: Extravascular mild chronic inflammation with broad and fibrotic hypodermal interlobular septum. Fascia over muscle is also broad and fibrotic with eosinophilic infiltration. Compatible with EF. M = Muscle, H: Hypodermis, F: Fascia. Hematoxylin and eosin (H&E) staining.

**FIGURE 5:**
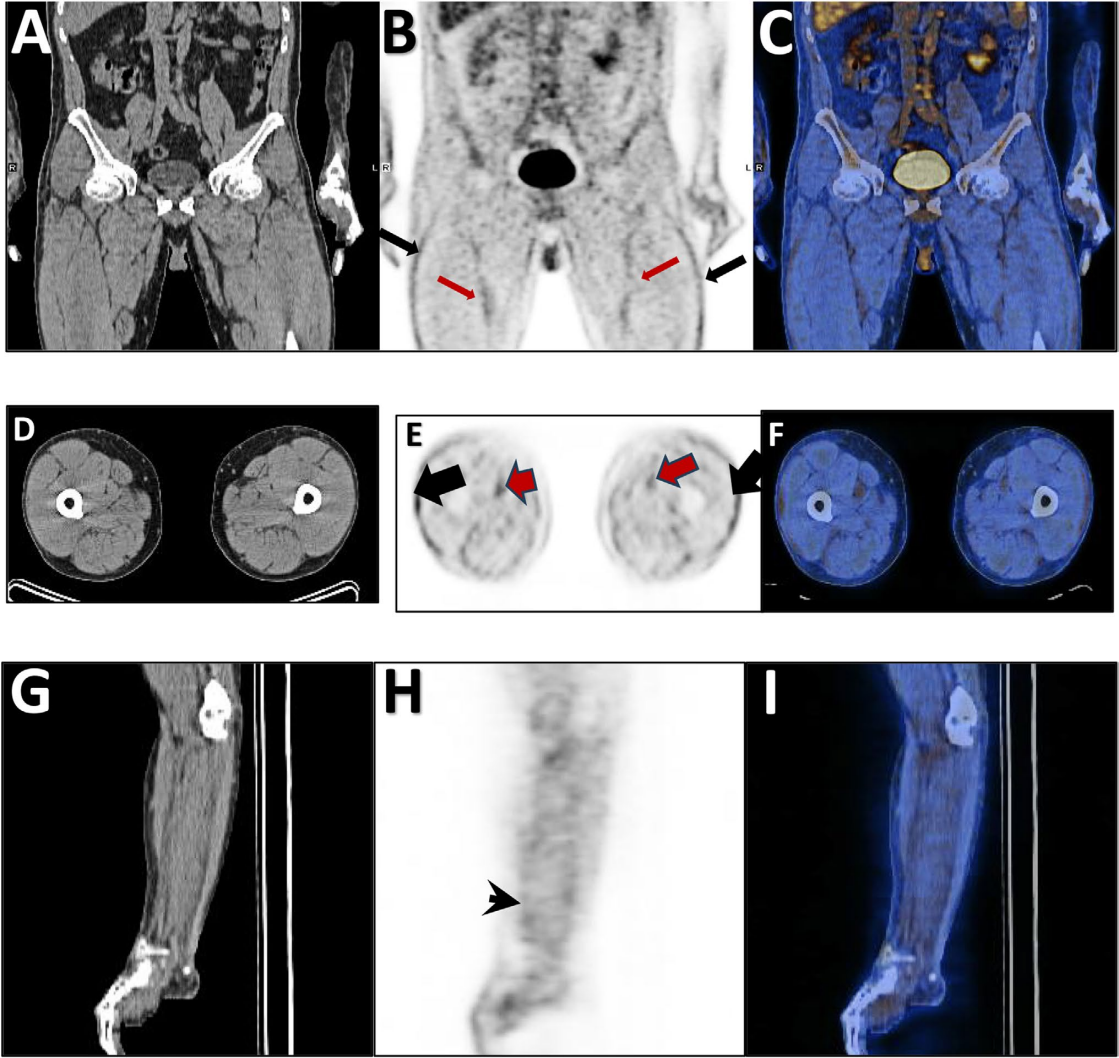
The 18F‐FDG PET/CT scan in coronal and axial views showed bilateral fascia‐lata fasciitis (black arrow in B, E); as well as intermuscular fasciitis (red arrow in B, E) (A‐F). Also, the PET/CT scan revealed medial and lateral intermuscular fasciitis (black arrow‐head in H, G‐I).

At the same time, the dermatologic manifestations appeared, and the skin of the inguinal area became indurated and painful. It extended upward in a symmetrical fashion, involving the abdomen and around the chest. Concurrently, skin hardening and tenderness of the forearms and the front of the thighs occurred with a peau d'orange appearance (Figure 1), resulting in a limited range of motion of elbows, and groove signs also appeared (Figure 2). Indurated skin around joints like the elbow and shoulder, as well as the pelvic area, made walking, moving, and daily routines so hard. The force of the limbs was normal on physical examination. However, skin thickness and stiffness were severe enough to impede chest expansion resulting in shortness of breath that was hard to tolerate. Due to the limitation in elbow movement, an elbow Magnetic Resonance Imaging (MRI) was performed for the patient, revealing edema in the subcutaneous tissue, particularly on the medial side of the elbow, extending to the proximal forearm (Figure 3). A rheumatology consultation was requested, and the patient was admitted to the rheumatology department with a suspicion of EF. A full‐thickness biopsy of the skin from the involved areas was performed, which confirmed EF (Figure 4). A few days later, progressive dyspnea accompanied by tachypnea began. Skin induration was severe and progressive, described as a band‐like pressure around the chest and abdomen, making breathing difficult. Additionally, urinary incontinence occurred due to the pressure exerted by the woody skin in the inguinal area. A positron emission tomography (PET) scan was also conducted, which showed diffuse symmetrical hypermetabolism in the deep fasciae, supporting the presence of metabolically active EF (Figure 5).

Due to the severity of the condition, the patient was treated with intravenous methylprednisolone at a dose of 1 g daily for 3 days, but this regimen did not yield a satisfactory response. Additionally, IVIG was administered at 5 g daily for 5 days. The patient was discharged on a regimen of prednisolone 50 mg once daily and methotrexate 15 mg weekly. After 2 months, methotrexate was discontinued due to lack of therapeutic response, and mycophenolate mofetil was initiated at a dose of 1 g every 12 h. Over the subsequent 6 weeks, prednisolone was tapered to 30 mg once daily.

### 2.3 Outcome and Follow‐Up

The patient was followed up 1 month later. Due to an unfavorable recovery, rituximab was initiated and repeated 2 weeks later, and then again 6 months later. Additionally, the patient received IVIG every 3 months. The skin in the abdominal area began to soften, although this improvement was incomplete. Following a dermatology consultation, PUVA (psoralen plus ultraviolet‐A radiation) therapy and topical tacrolimus were added to the treatment regimen. Currently, the patient is being managed with low‐dose corticosteroids, rituximab (every 6 months), and IVIG (every 3 months). After 12 months of follow‐up, a partial response to treatment was observed, indicating a relative improvement.

## 3 Discussion

EF is a rare autoimmune disorder that can be debilitating. Although the clinical, biological, and histological characteristics of EF are well‐defined, the optimal treatment approach remains uncertain [6]. Standard treatments, such as corticosteroids and immunosuppressive drugs, are effective for many patients; however, some cases are resistant to treatment, posing a significant challenge. This case report suggests that rituximab, an anti‐CD20 monoclonal antibody, may be an effective treatment option for EF that doesn't respond to standard therapies. The cases of rituximab treatment for EF that did not respond to other treatments are listed in Table 2 [2, 3, 5, 7, 8, 9, 10].

**TABLE 2:** Reported cases of the treatment of refractory cases of EF with rituximab.

Author et al.	Age onset/Sex	Clinical manifestations	Eosinophilic count	Prior treatments	RTX
Nikolaos Kougkas et al. 2021 [5]	58/F	edematous and indurated skin, peau d'orange	600/mm^3^	GCS/HCQ/AZA/MTX	Yes
Nikolaos Kougkas et.al. 2021 [5]	51/F	arthralgia, edematous and indurated skin, peau d'orange	800/mm^3^	GCS/CsA/MTX/AZA	Yes
Nikolaos Kougkas et.al. 2021 [5]	64/M	thick, edematous, and indurated skin, peau d'orange, arthritis	800/mm^3^	GCS/MTX	Yes
Erez Danial 2021 [3]	39/M	Swelling and indurated skin	> 3000/mm^3^	GCS/MTX/IVIG	Yes
Chan et al. 2018 [7]	17/M	edematous and indurated skin	NS	GCS/MTX/ AZA/MMF, CYC/CsA	No
Nahhas et al. 2018 [8]	67/M	myalgia, indurated skin	NS	Photopheresis/ MMF/GCS/ IVIG/HCQ/ CYC/Phototherapy	Yes
Thomson et al. 2015 [2]	41/M	Symmetrical forearm and calf pain and arthralgia	NS	GCS/MTX/ ETN/GOL	No
De Masson et al. 2013 [9]	57/M	asthenia, myalgia, and rapidly progressive thickening of the skin	2600/mm^3^	GCS/ATG/CsA	Yes
Scheinberg et al. 2006 [10]	20/NS	indurated skin	NS	NS	Yes

Abbreviations: ATG, anti‐thymocyte globulin; AZA, azathioprine; CsA, cyclosporine‐A; CYC, cyclophosphamide; ETN, etanercept; F, Female; GCS, glucocorticoids; GOL, Golimumab; HCQ, hydroxychloroquine; IVIG, intravenous immunoglobulin; M, Male; MMF, mycophenolate mofetil; MTX, methotrexate; NS, not specified; RTX, Rituximab.

EF is a rare autoimmune disease first described by Shulman in 1974 as “diffuse fasciitis with eosinophilia.” Patients with this condition often have a history of intense exercise or childbirth occurring a few days to 1–2 weeks before the onset of symptoms [11, 12]. The primary symptoms include symmetrical swelling with full circumference and stiffness of the plate‐like distal extremities, accompanied by redness and pain in the early stages [13]. Many cases are also accompanied by systemic symptoms, such as fever or general fatigue. The observed lesions extend to the proximal limbs but never affect the face or fingers. Full‐thickness skin biopsies reveal significant fascial thickening and infiltration of inflammatory cells, including lymphocytes and plasma cells. Eosinophilic infiltration is helpful for diagnosis but is typically observed only in the early stages of the disease [14, 15].

Refractory cases of EF are particularly challenging due to the risks associated with long‐term steroid use and the progressive nature of the condition, which can lead to contractures, chronic pain, and disability. In such cases, alternative therapeutic options are necessary [16]. Rituximab, a chimeric monoclonal antibody targeting CD20, a surface marker expressed on pre‐B and mature B lymphocytes, has a mechanism of action that involves the depletion of B cells through complement‐dependent cytotoxicity, antibody‐dependent cell‐mediated cytotoxicity, and direct induction of apoptosis [17, 18]. Rituximab has revolutionized the treatment of B‐cell‐driven autoimmune diseases, such as rheumatoid arthritis, systemic lupus erythematosus, and certain vasculitides. RTX has been used successfully in systemic sclerosis (SS), especially skin fibrosis [19, 20]. In recent years, its off‐label use has expanded to other autoimmune and inflammatory conditions, including EF. The rationale for using rituximab in EF is based on the involvement of B cells in autoimmune pathogenesis. B cells produce autoantibodies and function as antigen‐presenting cells, activating T cells and perpetuating the inflammatory response [3, 21]. By depleting B cells, rituximab may interrupt this immune cascade, thereby reducing inflammation and tissue fibrosis. Moreover, rituximab has demonstrated efficacy in conditions with overlapping features of EF, such as scleroderma and other fibrosing disorders, providing further justification for its use [22]. In the present case, the patient exhibited a refractory course of EF despite high‐dose corticosteroids and methotrexate.

IVIG is a plasma‐derived product containing immunoglobulins specifically IGG, with an immunomodulating effect on autoimmune diseases. It is particularly useful in infectious conditions where immunosuppressive agents cannot be administered to patients and in refractory cases that exhibit an inadequate response to initial immunosuppressive agents, including EF [23, 24]. Successful outcomes have been observed in studies involving refractory fasciitis, where IVIG facilitated the discontinuation of corticosteroids. The decision to initiate rituximab, IVIG, and mycophenolate mofetil was made after careful consideration of the disease's unresponsiveness to conventional therapies. Following treatment with rituximab and IVIG, the patient experienced clinical improvement in skin stiffness, pain, and functional limitations. This response was sustained over a period of several months, and no significant adverse events were associated with the use of rituximab. This favorable outcome emphasizes the potential role of rituximab and IVIG in managing refractory cases of EF.

This case report highlights the use of rituximab and IVIG as a treatment option for refractory EF. While our findings offer insights into the efficacy of rituximab and IVIG in this challenging context, several critical issues remain unresolved. The timing of when treatment begins is crucial in managing refractory EF. It remains unclear whether starting treatment earlier leads to better outcomes or if a more conservative approach could mitigate the risks associated with treatment. Additionally, an important question persists: are the observed improvements genuinely due to the therapeutic interventions, or could they simply result from the disease's natural progression? Another concern is the appropriateness of aggressive treatment strategies, including combination therapies. Was this approach justified in our case, or might a more patient‐centered and cautious strategy be more beneficial in reducing the risk of infection? Furthermore, the median time frame for clinical response to such therapies is unclear, which complicates treatment planning and patient counseling. It is essential to address these unanswered questions through larger studies and long‐term follow‐up to enhance our understanding and improve outcomes for patients with refractory EF. We hope that future research will clarify these critical aspects and ultimately lead to more effective, evidence‐based management strategies.

## Author Contributions

**Maryam Sahebari:** conceptualization, project administration, supervision, validation, visualization. **Motahhareh Karimoddini:** conceptualization, data curation, investigation, methodology, resources, software, writing – original draft, writing – review and editing. **Naser Tayyebi Meibodi:** investigation, visualization. **Sajad Ataei Azimi:** data curation, investigation, project administration, visualization. **Shadan Tafreshian:** conceptualization, data curation, investigation, methodology, resources, software, writing – original draft, writing – review and editing.

## Funding

The authors have nothing to report.

## Consent

“I provide my written authorization for the use of my clinical data in publications, with the understanding that this information will be utilized solely for educational purposes aimed at healthcare professionals.” Written informed consent was obtained from the patient to publish this report in accordance with the journal's patient consent policy.

## Conflicts of Interest

I declare that there are no conflicts of interest related to the publication of this manuscript. As the corresponding author and representative of all co‐authors, I confirm that the details furnished in this disclosure are accurate and comprehensive, to the best of my knowledge and belief.

## Data Availability

The data underpinning the findings of this study are available from the corresponding author upon reasonable request. Public availability of the dataset is limited due to privacy and ethical concerns.
